# Synthesis of Microscopic Cell Images Obtained from Bone Marrow Aspirate Smears through Generative Adversarial Networks

**DOI:** 10.3390/biology11020276

**Published:** 2022-02-10

**Authors:** Debapriya Hazra, Yung-Cheol Byun, Woo Jin Kim, Chul-Ung Kang

**Affiliations:** 1 Department of Computer Engineering, Jeju National University, Jeju 63243, Korea; debapriyah@jejunu.ac.kr; 2 Department of Computer Engineering, Jeju National University, Institute of Information Science & Technology, Jeju 63243, Korea; 3 Department of Laboratory Medicine, EONE Laboratories, Incheon 22014, Korea; oojinkim@eonelab.co.kr; 4 Department of Mechatronics Engineering, Jeju National University, Jeju 63243, Korea

**Keywords:** generative adversarial networks, microscopic cell images, bone marrow aspirate smears, synthetic images, classification

## Abstract

**Simple Summary:**

This paper proposes a hybrid generative adversarial networks model—WGAN-GP-AC—to generate synthetic microscopic cell images. We generate the synthetic data for the cell types containing fewer data to obtain a balanced dataset. A balanced dataset would help enhance the classification accuracy of each cell type and help with an easy and quick diagnosis that is critical for leukemia patients. In this work, we combine images from three datasets to form a single concrete dataset with variations of multiple microscopic cell images. We provide experimental results that prove the correlation between the original and our synthetically generated data. We also deliver classification results to showcase that the generated synthetic data can be used for real-life experiments and the advancement of the medical domain.

**Abstract:**

Every year approximately 1.24 million people are diagnosed with blood cancer. While the rate increases each year, the availability of data for each kind of blood cancer remains scarce. It is essential to produce enough data for each blood cell type obtained from bone marrow aspirate smears to diagnose rare types of cancer. Generating data would help easy and quick diagnosis, which are the most critical factors in cancer. Generative adversarial networks (GAN) are the latest emerging framework for generating synthetic images and time-series data. This paper takes microscopic cell images, preprocesses them, and uses a hybrid GAN architecture to generate synthetic images of the cell types containing fewer data. We prepared a single dataset with expert intervention by combining images from three different sources. The final dataset consists of 12 cell types and has 33,177 microscopic cell images. We use the discriminator architecture of auxiliary classifier GAN (AC-GAN) and combine it with the Wasserstein GAN with gradient penalty model (WGAN-GP). We name our model as WGAN-GP-AC. The discriminator in our proposed model works to identify real and generated images and classify every image with a cell type. We provide experimental results demonstrating that our proposed model performs better than existing individual and hybrid GAN models in generating microscopic cell images. We use the generated synthetic data with classification models, and the results prove that the classification rate increases significantly. Classification models achieved 0.95 precision and 0.96 recall value for synthetic data, which is higher than the original, augmented, or combined datasets.

## 1. Introduction

Microscopic images are considered to be an essential and gold standard in the determination and diagnosis of multiple diseases [1]. Bone marrow is a spongy tissue found in the center of the bone containing immature cells called stem cells that produce blood cells. The main task of bone marrow is to produce red blood cells, white blood cells, and platelets [2]. Biopsy of bone marrow includes the collection of bone marrow samples as well as examining the cells, their structure, and counts under the microscope [3]. Bone marrow aspiration is a collection of fluid-containing cells that can be evaluated or examined under a microscope. The slides of stained smears are examined microscopically by specialists, and the cells are evaluated based on the number, appearance, type, etc. Abnormal cellular components indicate the presence of blood diseases. For example, excess granulocytes such as myeloblasts refer to the presence of acute myeloid leukemia [4].

The representation and classification of individual cells have been performed in various histopathology works such as lung cancer, breast cancer, brain cancer, colon cancer, acute leukemia, and many more. Not only classification, but also medical image analysis include the automatic detection of tumors, localization of tumors or cancerous cells, and the early prediction of deadly diseases. Generative adversarial network (GAN) [5] is the emerging framework and has gathered much attention in the medical image analysis domain. Deep learning has been proven to be a powerful tool in modern medical diagnosis and histopathology image analysis [6,7]. GAN has the potential to transform random noise variables into visually realistic images by learning the original data distribution. Initially, the training of Vanilla GAN was unstable. Wasserstein GAN (WGAN) [8], and Wasserstein GAN with gradient penalty (WGAN-GP) [9] has considerably improved the training process of GAN. Conditional GAN (CGAN) was introduced later, where the GAN architecture could be conditioned with special cases. Until this time, the utility of the GAN architecture has increased enormously. Medical image analysis requires abundant data for enhancing the accuracy of machine learning models since, in real-time, there is no room for incorrect results in the medical domain. But on the other hand, it is important to keep patient confidentiality intact. Therefore, there is a need to generate synthetic data that are realistic and maintain the original data distribution.

Hüseyin et al. proposed a framework to automatically detect and classify white blood cells using regional convolutional neural networks [10]. The authors classified five types of white blood cells. The dataset was manually labeled, and variations of R-CNN [11], fast R-CNN [12], and faster R-CNN [13] were used to classify the dataset. Xie et al. proposed a methodology that learns the representation of features and assignment of clusters simultaneously through deep neural networks and calls it deep embedded clustering that is applicable on images [14]. Zhang et al. proposed a probabilistic-based hashing technique for multiple cues of cell-level analysis [15]. In recent work, Ching-Wei Wang et al. introduced a fully automatic bone marrow whole slide image analysis based on deep learning for cell detection and classification [16].

Li Ma et al. proposed a combination of DCGAN [17], and ResNet for blood cell image classification [18]. The authors introduced a new loss function that has improved the discriminative architecture of the GAN model. Shuaizhen et al. developed a weighted feature transfer GAN to synthesize unpaired multi-model medical images accurately [19]. The adversarial loss is combined with the feature map to make meaningful local features of the medical images. Gozes et al. generated synthetic patches of mitosis for enhancing classification of cell images using GAN [20]. In another work, Halicek et al. implemented a conditional GAN for the synthetic generation of hyperspectral cell im-ages of breast cancer obtained from digital histology [21]. DermGAN incorporates the pix2pix architecture to generate synthetic data for clinical skin images [22]. The author also tested the model with a skin condition classifier for classifying malignant conditions. The modified generator GAN MG-GAN was introduced by Poonam et al. to augment medical data for the improvement of cancer classification [23].

In this paper, we propose a hybrid GAN architecture that inherits the supremacy of both WGAN-GP and auxiliary classifier GAN (AC-GAN) [24]. The main contribution of this paper is as follows:

- We have prepared a new dataset that consists of microscopic cell images obtained from bone marrow aspirate smears collected from three different data sources. The dataset has been prepared with the help of experts from the relevant field;
- We present a GAN model WGAN-GP-AC that uses the WGAN-GP model combined with the architecture of AC-GAN to generate synthetic microscopic cell images obtained from bone marrow aspirate smears;
- We use the synthetic data for classification purposes and provide results comparing the performance of classification models using synthetic and original data.

## 2. Dataset

We collected datasets from three sources and combined them to form a complete dataset for this work. Our main source of data was EONE Laboratories [25] in South Korea. EONE Laboratories provided microscopic images of 17 cell types collected from bone marrow aspirate smears. The dataset contained a total of 12,756 images of individual cells of patients with and without blood diseases. The first dataset contained the following cell types: basophil, eosinophil, neutrophil band, neutrophil segmented, lymphocyte atypical, lymphocyte typical, immature granulocytes, monocytes, erythroblasts, platelets, myelocyte, myeloblast, smudge cells, metamyelocyte, promyelocyte bilobed, promyelocyte and monoblast. Our second data source was from Mendeley data [26] which contained microscopic peripheral blood cell images. The second dataset contained 10,122 individual cell images, which was obtained using the CellaVision DM96 analyzer in the hospital clinic of Barcelona, Core Laboratory. The cell types in this dataset are basophil, eosinophil, neutrophil, lymphocytes, immature granulocytes, monocytes, erythroblasts and platelets. Each image was of size 360 × 360 pixels in JPG format and was annotated by experts in the field. These images were acquired from individuals without any infection or oncologic and hematologic disease. The individuals were also free from any drug consumption for any treatment. Our third data source was the cancer imaging archive [27]. This dataset was prepared by Munich University Hospital and contained 10,300 single cell images labeled by experts and procured from bone marrow aspirate smears. The data were collected from 100 patients diagnosed with acute myeloid leukemia and from 100 patients who had blood disease but were not malignant. The third dataset contained 15 cell types: basophil, eosinophil, erythroblast, smudge cells, lymphocyte atypical, lymphocyte typical, metamyelocyte, monoblast, monocyte, myelocyte, myeloblast, neutrophil band, neutrophil segmented, promyelocyte bilobed, and promyelocyte.

**Figure 1:**
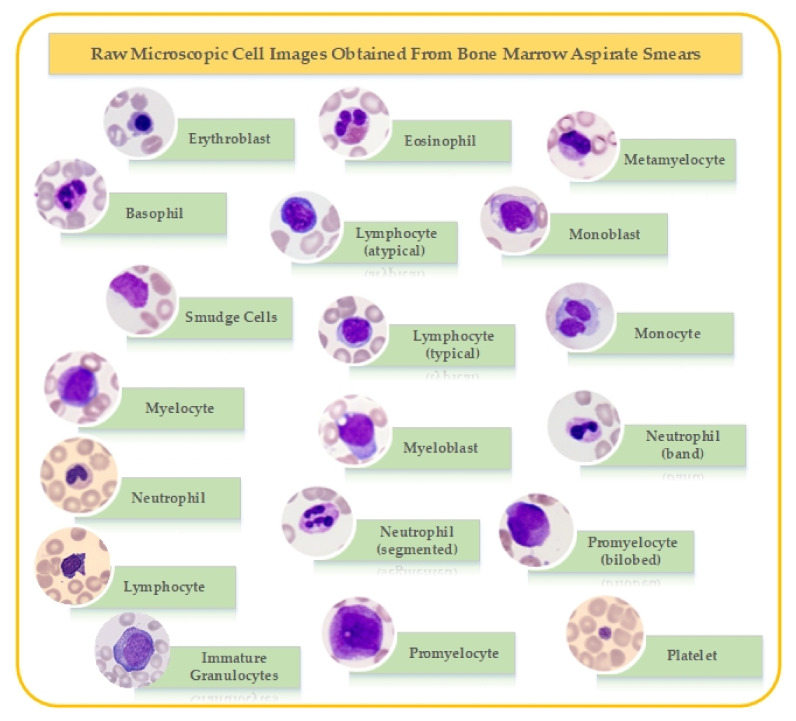
Sample example of types of microscopic cell images in the dataset.

Figure 1 shows sample examples of how each cell types look in microscopic images. In total, the dataset was combined to form 19 cell types.

## 3. Methodology

In this section, we describe the total implementation process followed to generate synthetic microscopic cell images acquired from bone marrow aspirate smears. As explained in the dataset section, we collected images from three data sources. The first part of the methodology is to collect data and combine the images that belong to the same class. We took the help of experts from EONE Laboratories first to combine the images belonging to the same group. The total number of cell types before preprocessing was eighteen. The cell types were basophil, eosinophil, erythroblast, immature granulocytes, lymphocyte, lymphocyte atypical, lymphocyte typical, metamyelocyte, monoblast, monocyte, myeloblast, myelocyte, neutrophil, neutrophil segmented, neutrophil band, platelet, promyelocyte, promyelocyte bilobed, and smudge cells. Counts per cell type are as mentioned in Table 1.

**Figure 2:**
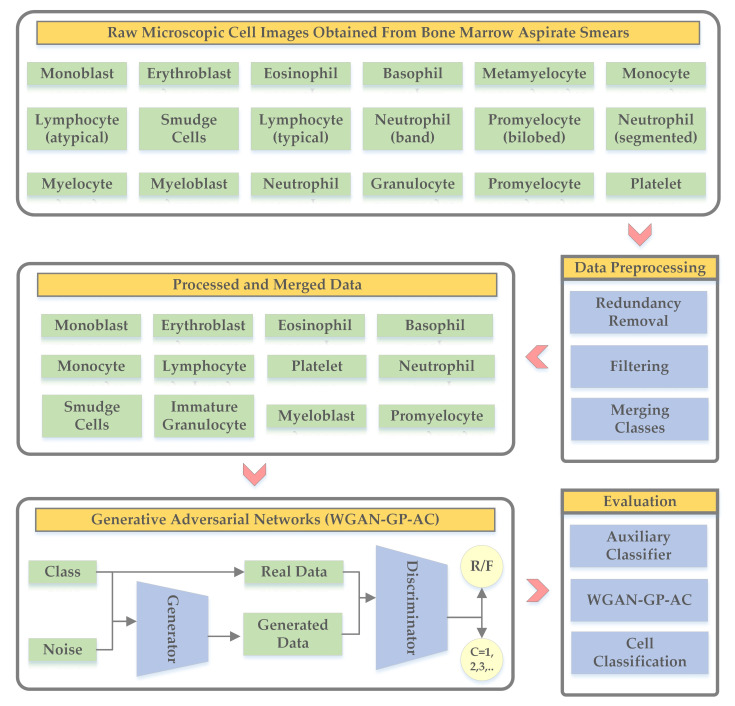
Overview of the proposed methodology to generate synthetic microscopic cell images obtained from bone marrow aspirate smears through generative adversarial networks.

The overall workflow is presented in Figure 2, where we combine the dataset from three sources and form a dataset with microscopic cell images belonging to 18 cell types. The next phase is data preprocessing which has been done with the help of experts in the medical field. After data preprocessing, we form the final dataset containing twelve classes or cell types.

The dataset is processed through our proposed GAN architecture. For the GAN architecture, we combine the loss function of WGAN-GP and discriminator architecture of auxiliary classifier GAN to form WGAN-GP-AC. The proposed GAN models generate synthetic microscopic individual cell images that machine learning algorithms can further use for classification, detection, and so forth. The generated images are evaluated based on their quality, classification accuracy, and similarity with the original images.

### 3.1. Data Preprocessing

In the data preprocessing section, redundant data or duplicate images were removed and images were filtered by experts from EONE Laboratories. Firstly, images from three sources belonging to similar cell types were combined. Then, redundant and incorrect data were eliminated through expert reviews. After elimination, images from cell types belonging to the same cell family were merged to form a single group or cell type. For example, lymphocyte, lymphocyte atypical, and lymphocyte typical were combined to form a single cell type, that is, lymphocyte.

Cell type neutrophil was formed by combining images belonging to neutrophil, neutrophil band, and neutrophil segmented. Promyelocyte was formed by merging images of promyelocyte and promyelocyte bilobed. Immature granulocyte contained images from immature granulocyte, myelocyte, and metamyelocyte. After performing all the preprocessing techniques, the total number of cell types was 12 with the following count as mentioned in Table 2.

We have used the stain normalization process so that there would be no bias during the network training. For the classification of each cell type, we have divided the datatset into 70% training, 30% testing and from the training dataset we have used 20% for validation.

### 3.2. WGAN-GP-AC

Generative adversarial networks (GAN) were introduced by Ian Goodfellow et al. [5] in the year 2014. GAN was proposed as a framework of unsupervised generative models comprising a generator and a discriminator network. GAN is widely used in various fields for different applications.

The generator network *G* in the GAN architecture is trained to generate synthetic samples, whereas the task of the discriminator *D* is to discriminate between original and generated samples. This is known as the minmax game where the generator tries to fool the discriminator by producing realistic samples and the discriminator tries to correctly identify real from fake. The generator takes in a random noise variable that retains the data distribution $P_{d}$ over original data distribution *x*. The generated samples are then passed to the discriminator along with the original samples. The generator tries to minimize log(1−D(G(z))) with the minmax function defined as in (1):

(1) $$\min_{G}\;\max_{D}\;f\left(D,G\right)=E_{x∼P_{d}\left(x\right)}\left[logD\left(x\right)\right]+E_{z∼P_{d}\left(z\right)}\left[log\left(1-D\left(G\left(z\right)\right)\right).\right]$$

Generally, the loss function of Vanilla GAN is used to train GAN, which makes the training process harder and the convergence rate slower. The main problem of training with the loss function of Vanilla GAN is that it leads to mode collapse problems. It becomes difficult to assess whether the generator is still in training, has collapsed, or is still converging. Therefore, in this work, we follow the work of Wasserstein GAN with gradient penalty (WGAN-GP) [3]. The Wasserstein GAN [2] solves the problems faced by Vanilla GAN by optimizing the Earth Mover distance, also known as the Wasserstein-1 distance, instead of the Jensen-Shanon divergence (JSD) that is used in the Vanilla GAN. WGAN builds a powerful discriminator that would generate a significant gradient even if the generator performs poorly. The Wasserstein-1 distance can be defined as in (2) with *D* being the k-Lipschitz function.

(2) $$\min_{G}\;\max_{D}\;E_{x∼P_{d}\left(x\right)}\left[D\left(x\right)\right]+E_{z∼P_{z}}\left[D\left(G\left(z\right)\right).\right]$$

**Figure 3:**
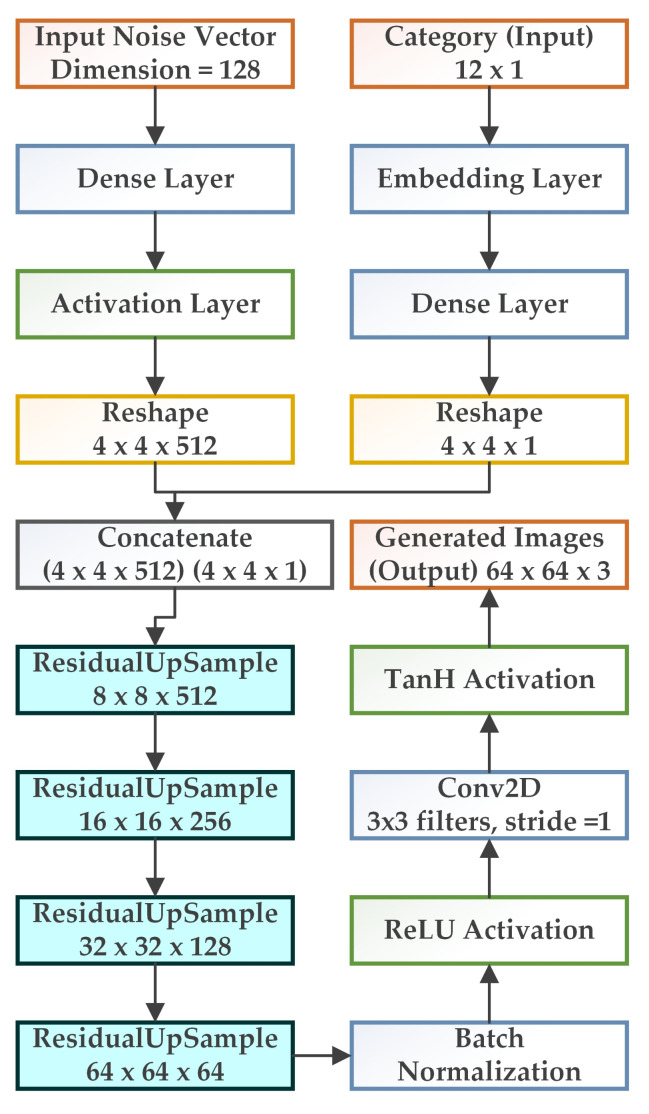
Generator architecture for the proposed GAN model.

WGAN requires critic weight-clipping in a compact space [−c,c] where the critic is considered the optimal discriminator. WGAN-GP is an improved version of WGAN proposed by Gulrajani et al. that replaces the weight clipping mechanism required in WGAN and complies with the condition of 1-Lipschitz by introducing the gradient penalty. The objective of WGAN-GP is a combination of the original critic loss and the gradient penalty as shown in (3) and (4):

(3) $$OriginalCriticLoss=E_{\tilde{x}∼P_{d}}\left[D\left(\tilde{x}\right)\right]-E_{x∼P_{r}}\left[D\left(G\left(x\right)\right)\right]$$

(4) $$GradientPenalty=\sigma E_{\hat{x}∼P_{\hat{x}}}[(∥\nabla_{\hat{x}}D\left(\hat{x}\right){∥}_{2}-1{)}^{2}],$$

where $\hat{x}∼P_{\hat{x}}$ defines random samples and $P_{d}$ and $P_{r}$ are data distribution and generator distribution. σ has been selected as 10 according to our data and experiments In our proposed work, the generator receives input noise vector and category or class labels as inputs, which are passed through the dense and activation layers. The images and the labels are then reshaped. After reshaping, it is concatenated and passed through residual upsampling blocks. After which, we perform batch normalization. The synthetic data are generated through Conv2D and tanh activation as shown in Figure 3.

For the discriminator, we use the concept of the auxiliary classifier GAN, which is different from the conditional GAN in the sense that the discriminator in the ACGAN does not receive class information. The task of the discriminator is to output the probability distribution of the image source (real or generated) and to output the probability distribution over the class labels for the particular image that is done by the auxiliary classifier in the discriminator architecture. Therefore, the objective function for the auxiliary classifier GAN is the combination of $S_{L}$ and $C_{L}$ , where $S_{L}$ is the correct source log-likelihood and $C_{L}$ is the log-likelihood of the correct cell type. $S_{L}$ and $C_{L}$ are defined as in (5) and (6):

(5) $$S_{L}=E\left[\log P\left(S=real|x_{real}\right)\right]+E\left[logP\left(S=synthetic|x_{synthetic}\right)\right]$$

(6) $$C_{L}=E\left[\log P\left(C=c|x_{real}\right)\right]+E\left[logP\left(C=c|x_{synthetic}\right)\right].$$

**Figure 4:**
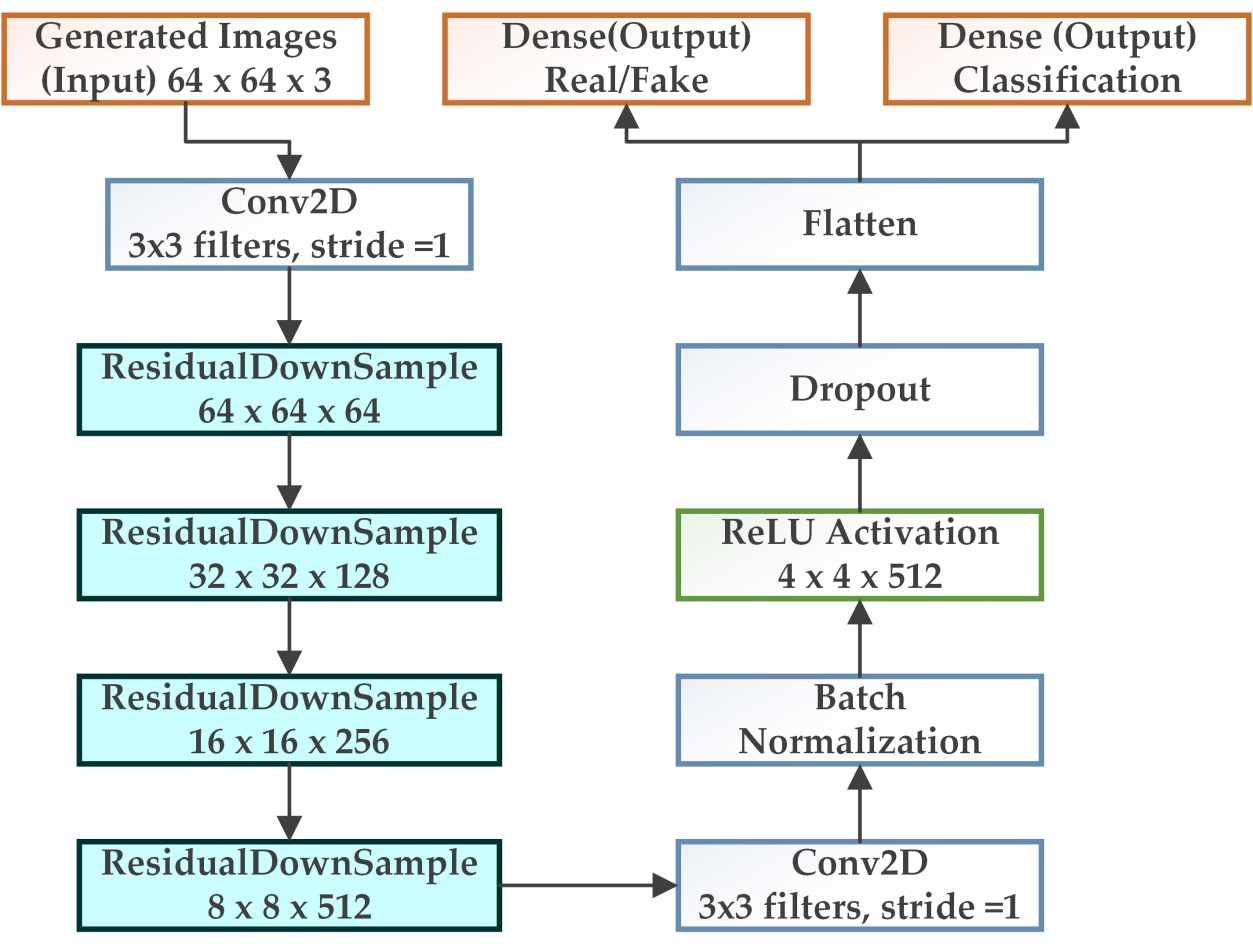
Discriminator architecture for the proposed GAN model.

Figure 4 describes the architecture of the discriminator of WGAN-GP-AC. As we can see from Figure 4, the discriminator is provided only with the generated images, but not with the class labels. The images pass through the 2D convolutional layer and residual downsampling blocks. The discriminator tries to maximize the probability of correctly classifying real and synthetic images and correctly predicting the cell types or class labels.

**Figure 5:**
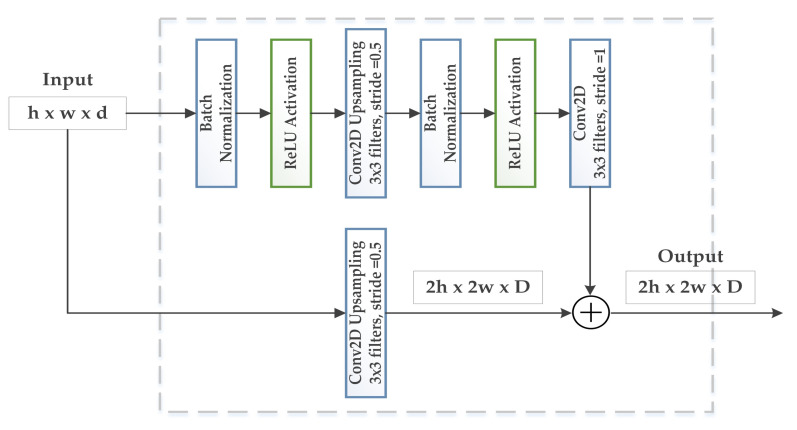
Illustration of residual block (upsampling) in the proposed architecture.

**Figure 6:**
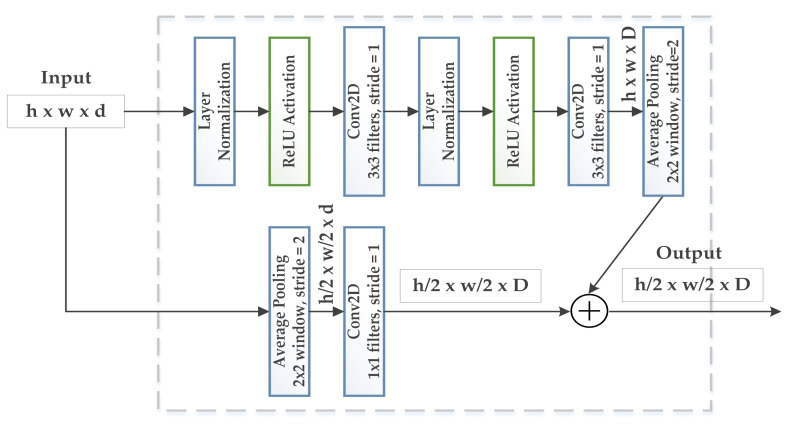
Illustration of residual block (downsampling) in the proposed architecture.

This way, the GAN model generates more realistic images that can be used for real-time classification models or any other machine learning algorithms. Figure 5 and Figure 6 present the residual block architecture for upsampling and downsampling.

## 4. Experiments and Results

This section defines the evaluation metrics we used to evaluate our model’s performance in generating synthetic microscopic cell images. The evaluation is done in three stages. We first measure how combining the auxiliary classifier’s concept with WGAN-GP performs compared to other existing GAN models. We also compute the training accuracy and loss of the proposed model and see the quality of the generated images. We used a learning rate of 0.001, batch size 64 and patch size as 128 × 128. We provide sample examples of generated images through the proposed WGAN-GP-AC model. We present the error rates of different architectures of GAN on our prepared dataset. Lastly, we use original and synthetic data separately to measure how classification models work. We compare the performance of pre-trained models on original and synthetic data.

The GAN architectures that we use to compare our modelâs performance are auxiliary classifier GAN (AC-GAN) [24], Wasserstein GAN (WGAN) [8], WGAN with gradient penalty (WGAN-GP) [9], information maximizing GAN (InfoGAN) [28], WGAN-GP-Info [29], deep convolutional GAN (DCGAN) [17] and conditional GAN (CGAN) [30].

In Table 3, we present a quantitative comparison of various models on our dataset. The evaluation metrics which we chose are inception score (IS), learned perceptual image patch similarity (LPIPS), recall, precision, F1 score, and Fréchet inception distance (FID). Through the inception score, we compute how realistic the generated images are. The formulation for the inception score is shown as in (7).

(7) $$e(E_{x}\left[KLD\left(p\left(y|x\right)\|p\left(y\right)\right)\right]),$$

where KLD is the Kullback–Leibler divergence measuring the difference between the marginal distribution p(y) and probability distribution of image × denoted by p(y|x) . The LPIPS metric is used to measure the variance in the synthetic samples generated by WGAN-GP-AC. Whereas FID computed the distance of the feature vector between real and synthetic images. It compares the distribution of the synthetic images along with the distribution of the real images used during training the generator. The lower the FID value, the higher the quality of the generated image. The following Equation (8) can be used to compute the FID between real images *R* and synthetic images *S*:

(8) $$FID=∥\mu_{R}-\mu_{S}{∥}^{2}+T_{R}(\Sigma_{R}+\Sigma_{S}-2{\left(\Sigma_{R}\Sigma_{S}\right)}^{1/2}.$$

Given the real and synthetic distribution, precision measures the quality of generated samples and indicates how accurately the auxiliary classifier is predicting the classes, whereas recall measures the quantity. The higher the value for precision and recall, the better the model’s performance. F1, on the other hand, is the harmonic mean between the precision and the recall and contributes to the measurement of the model’s accuracy on a particular dataset. The experiment result shows that our proposed model WGAN-GP-AC performs better than the other mentioned existing models and improves the quality of multiclass image generation as compared to WGAN-GP and AC-GAN individually.

**Figure 7:**
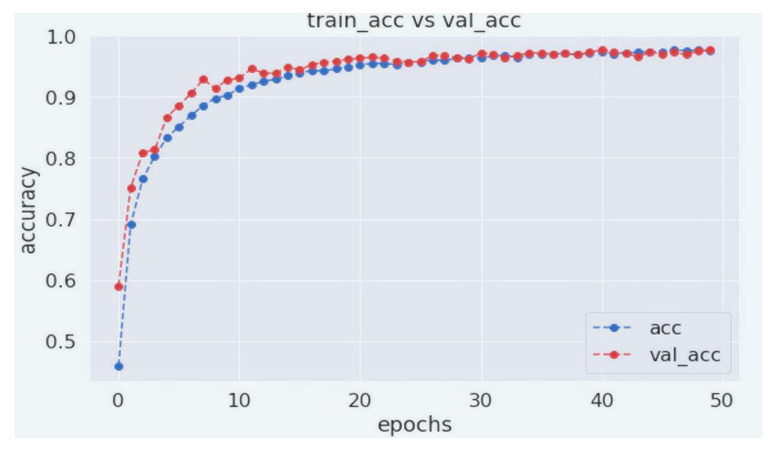
Training and validation accuracy of WGAN-GP-AC.

**Figure 8:**
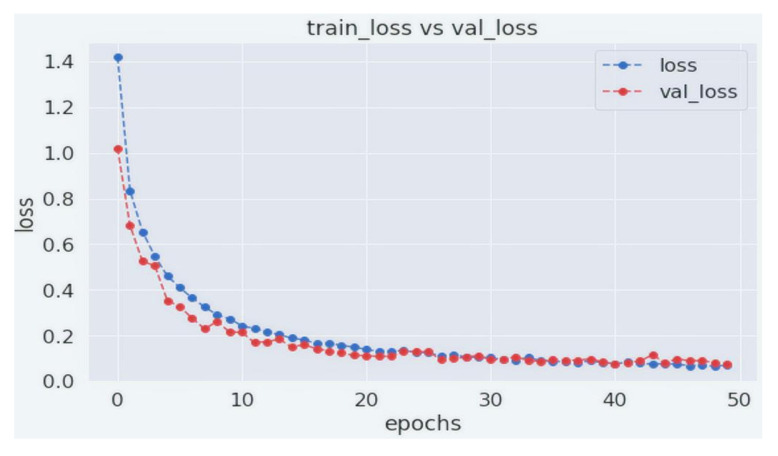
Training and validation loss of WGAN-GP-AC.

Figure 7 and Figure 8 show the training and validation accuracy and loss. As can be seen from the figures, our proposed WGAN-GP-AC model produces a consistent performance after 25 epochs. Our model is trained for 50 epochs with 265 iterations per epoch. As can be seen, the model requires less computational overhead to perform significantly better. It achieves a training accuracy of 97.54% and a validation accuracy of 97.32%. Training loss for our model was 0.0692, with a validation loss of 0.0917.

Table 4 presents the comparison of error rates (l1 and l2), peak signal-to-noise ratio (PSNR), and structural similarity index metric (SSIM). The loss functions l1 and l2 are important evaluation metrics for measuring the error rates.

The least absolute deviation is measured by l1 and can be computed to minimize error, which is defined as the sum of all the absolute differences between original and synthetic data as shown in (9):

(9) $$l1=\Sigma_{i=1}^{n}\left|y_{real}-y_{synthetic}\right|$$

(10) $$l2=\Sigma_{i=1}^{n}{\left(y_{real}-y_{synthetic}\right)}^{2}$$

l2, as defined in (10), is another loss function used in GAN to measure error, that is, the squared differences between real and synthetic data. We measure both l1 and l2 errors to test our model with outliers, if any. l1 and l2 are the measure for reconstruction error between the synthetic and the real images. PSNR and SSIM are image quality measures. PSNR computes the peak signal-to-noise ratio between the real and synthetic images. The higher the PSNR, the better is the quality of the synthetic image. Mean squared error (MSE) is the cumulative squared error between the generated and the real image, whereas PSNR computes the peak error. PSNR can be calculated from Equations (11) and (12) where *M* and *N* are total rows and columns in the images and *R* is the maximum possible pixel value of the image.Results show that our proposed model generates less error as compared to other models. WGAN-GP-AC also produces a higher PSNR value and a high structural similarity index, which indicates that the synthetic image generated by our proposed model is of greater quality than other existing models. A crucial aspect of generating synthetic data is to evaluate whether machine learning models can use it for real-life experiments such as classification.

(11) $$\mathrm{MSE}=\frac{\sum_{M,N}{\left[real(m,n)-synthetic(m,n)\right]}^{2}}{M*N}$$

(12) $$\mathrm{PSNR}=10\log_{10}\left(\frac{R^{2}}{MSE}\right)$$

SSIM uses three quantities, that is, luminance (*L*), contrast (*C*), and structure (*S*) to measure the corresponding pixels and their neighbors in real and synthetic images. *L*, *C* and *S* can be defined as in Equations (13)–(15), where μ and σ denotes mean and standard deviation and $C_{1}$ , $C_{2}$ and $C_{3}$ are constants included for numerical stability. SSIM can be defined as in (16).

(13) $$L\left(real,synthetic\right)=\frac{2\mu_{real}\mu_{synthetic}+C_{1}}{\mu_{real}^{2}+\mu_{synthetic}^{2}+C_{1}}$$

(14) $$C\left(real,synthetic\right)=\frac{2\sigma_{real}\sigma_{synthetic}+C_{2}}{\sigma_{real}^{2}+\sigma_{synthetic}^{2}+C_{2}}$$

(15) $$S\left(real,synthetic\right)=\frac{\sigma_{realsynthetic}+C_{3}}{\sigma_{real}\sigma_{synthetic}+C_{3}}$$

(16) SSIM(real,synthetic)=L(real,synthetic)*C(real,synthetic)*S(real,synthetic).

In Table 5 and Table 6, we have presented the performance of transfer learning models such as InceptionV3 [31], ResNet [32], VGG16 [33], CNN [34], Xception [35] and VGG19 [36] to measure how accurately the classifiers can classify the cell types after training it with original and synthetic data separately. We have used precision and recall as evaluation metrics. For precision, recall and F1, we have used the computational method as mentioned in [37,38]. The results show that the accuracy of the models is enhanced when synthetic data generated by WGAN-GP-AC are used to train the model.

We have also compared the performance of classification models using original, synthetic, augmented data and a combination of original and synthetic data. For augmentation, we have used three different combinations of augmentation techniques. Augmentation-1 uses scaling, rotation, and color augmentation techniques; Augmentation-2 uses translation, contrast, and scaling methods; and Augmentation-3 uses saturation, scaling, and rotation. We have kept the total number of images for each cases same so that there is a fair comparison.

We have first trained the classification models with original data and also tested the models with original data. The training and testing ratios were 70% and 30%. For validation, we used 20% of the training data. We then followed the same process to get the performance of the classification models with synthetic data, augmented data and mix of original and synthetic data. As can be seen from all the results, the images generated by our proposed WGAN-GP-AC model are of better quality and it performs better, quantitatively, than other GAN models.

**Figure 9:**
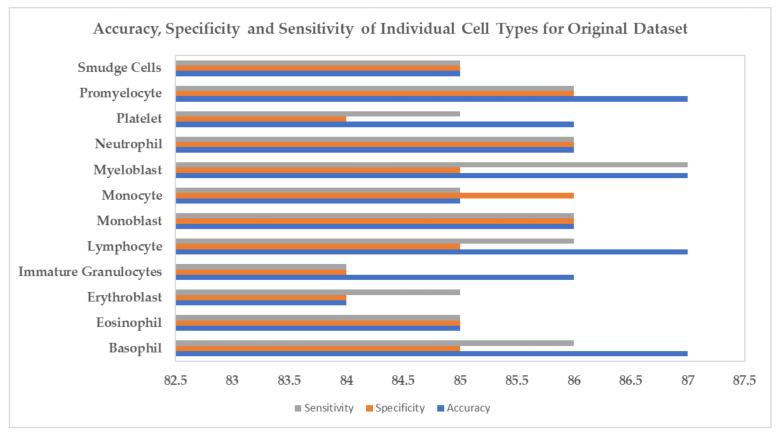
Accuracy, Specificity and Sensitivity of Individual Cell Types for Original Dataset.

**Figure 10:**
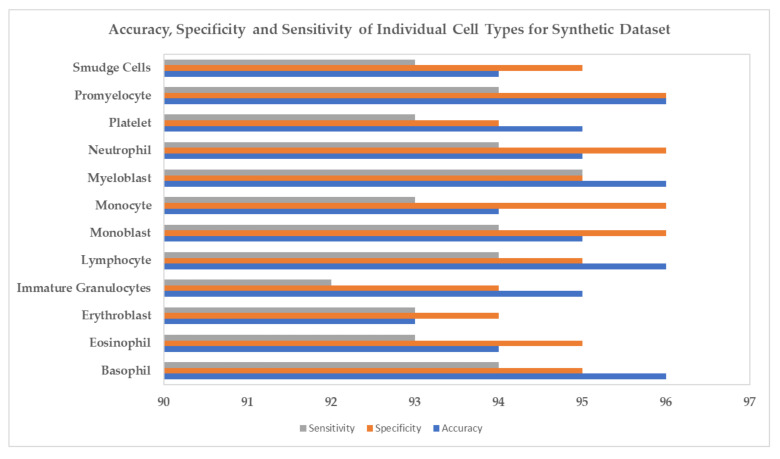
Accuracy, Specificity and Sensitivity of Individual Cell Types for Synthetic Dataset.

**Figure 11:**
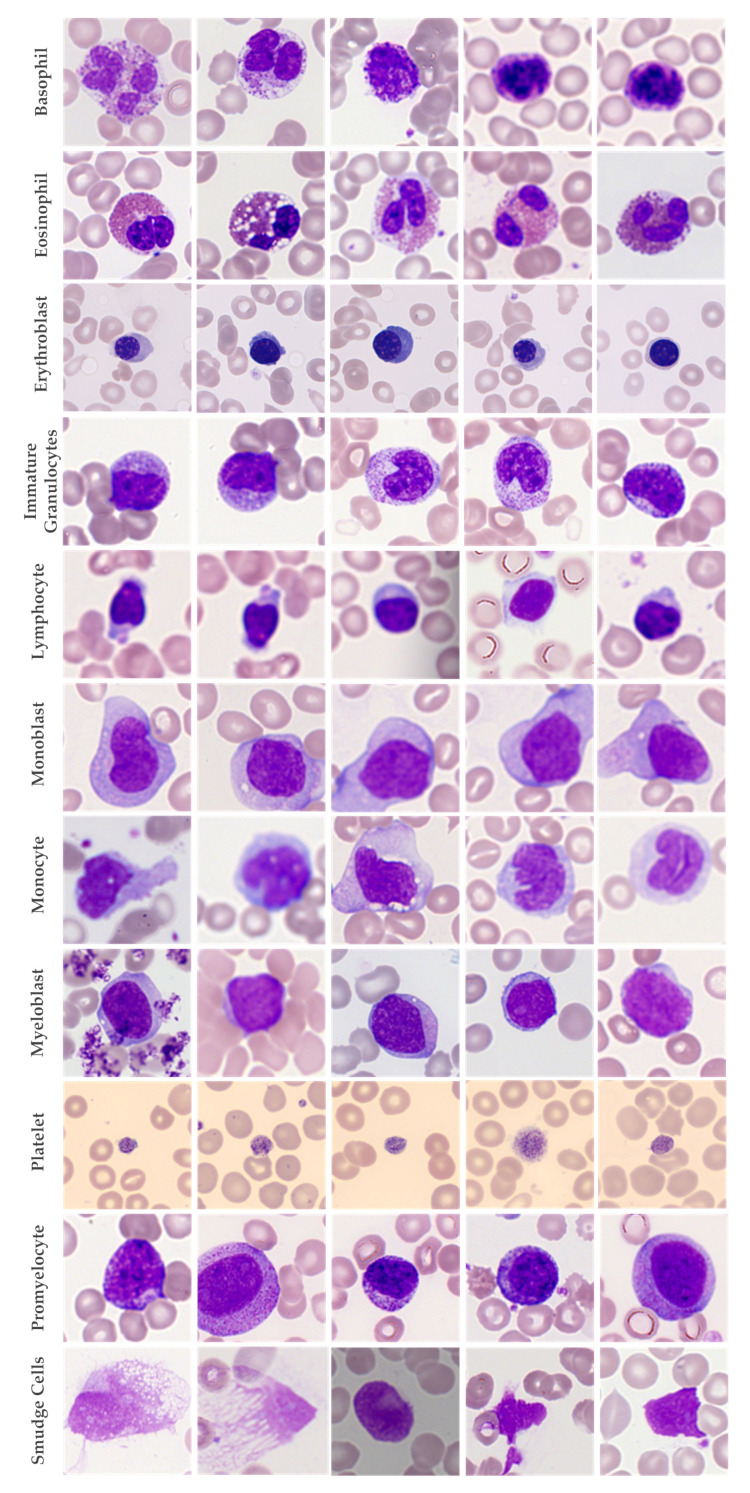
Sample images generated for each cell type by WGAN-GP-AC.

In Figure 9 and Figure 10, we have shown each cell type’s accuracy, specificity, and sensitivity for the InceptionV3 classification model using the original and synthetic datasets separately. We present examples of the generated synthetic microscopic images for each cell type in Figure 11.

## 5. Conclusions and Discussion

In this work, we propose a hybrid GAN architecture that implements the concept of auxiliary classifier GAN in Wasserstein GAN with gradient penalty. We use the loss function of WGAN-GP but implement the discriminator as in the AC-GAN. The discriminators’ task in this work is not only to identify real or fake but also to assign or classify classes, that is, cell types of each image. This architecture helps generate synthetic microscopic bone marrow aspirate smears cell images that can be used for multiclass classification in real life. It enhances the classification results by oversampling the minority classes (i.e., cell types with fewer images) and balancing the dataset.

We first take microscopic images of 19 cell types obtained from bone marrow aspirate smears, which are then preprocessed. In our work, we collaborated with experts to filter data, rearrange images to proper classes if they were marked with wrong cell types, we merged cell types belonging to the same cell family. After preprocessing, the cell types were reduced to 12. Every image of each cell type is processed through our proposed WGAN-GP-AC model. The generator produces synthetic data, and the discriminator evaluates the images as real or fake and tries to classify the cell type for every image. The generated images are evaluated through various evaluation metrics. Our GAN model obtains training and validation accuracy of 97.54% and 97.32%. The quantitative result shows that our model generates less error, has more structural similarity with the original data, and produces better quality images for every cell type as compared to other GAN models. We have included the results for different augmentation techniques and the dataset prepared through a combination of original and synthetic data. We used the same number of images for synthetic, augmented, and a combination of the original and synthetic datasets to evaluate the classification models. The result shows that the classification models perform better for the synthetic dataset generated by our proposed model. We provide accuracy, specificity, and sensitivity scores for each cell type in this work. We provide the results for the classification of each cell type using the original and synthetic datasets separately. We also experiment our data with different classifiers, which indicates that the accuracy of classification models increases while using synthetic data generated from WGAN-GP-AC. In the future, we would like to implement a balancing mechanism that would oversample minority classes or cell types with less images through GAN and undersample majority classes through image similarity measures so that the classification accuracy could enhance their performance. We also plan to generate images with multiple cell types in a single image so that they can be used for disease diagnosis, early detection, and other medical reasons.

## Figures and Tables

**Table 1:** Number of images per cell type before preprocessing.

Cell Type	Number of Images	Dataset-1	Dataset-2	Dataset-3
Basophil	1224	570	420	234
Eosinophil	3538	1061	1356	1121
Erythroblast	1547	540	500	507
Immature Grannulocytes	2881	1266	1615	N/A
Lymphocyte	1213	N/A	1213	N/A
Lymphocyte Atypical	7	4	N/A	3
Lymphocyte Typical	3818	1790	N/A	2028
Metamyelocyte	13	8	N/A	5
Monoblast	26	14	N/A	12
Monocyte	2583	912	1013	658
Myeloblast	3104	1246	N/A	1858
Myelocyte	39	22	N/A	17
Neutrophil	3316	N/A	3316	N/A
Neutrophil Band	82	42	N/A	40
Neutrophil Segmented	7346	3588	N/A	3758
Platelet	2339	1650	689	N/A
Promyelocyte	69	26	N/A	43
Promyelocyte Bilobed	18	10	N/A	8
Smudge Cells	15	7	N/A	8

**Table 2:** Number of images per cell type after preprocessing.

Cell Type	Number of Images
Basophil	1224
Eosinophil	3538
Erythroblast	1547
Immature Grannulocytes	2933
Lymphocyte	5038
Monoblast	26
Monocyte	2583
Myeloblast	3104
Neutrophil	10,743
Platelet	2339
Promyelocyte	87
Smudge Cells	15

**Table 3:** Quantitative comparison on our dataset.

Models	IS	FID	LPIPS	Precision	Recall	F1
AC-GAN	8.34 ± 0.89	76.3	0.34	94.37	94.01	94.13
WGAN	9.67 ± 0.25	72.3	0.31	94.58	94.57	93.06
WGAN-GP	10.06 ± 0.03	71.1	0.29	96.72	95.38	95.11
InfoGAN	9.12 ± 0.37	73.9	0.32	94.01	94.83	94.92
WGAN-GP-Info	9.94 ± 0.71	73.1	0.33	94.49	95.03	94.02
DCGAN	9.89 ± 0.28	73.4	0.31	95.66	94.91	95.50
CGAN	9.01 ± 0.77	75.2	0.34	93.01	93.48	92.99
WGAN-GP-AC	**12.36 ± 0.41**	**67.2**	**0.25**	**96.83**	**96.09**	**96.32**

**Table 4:** l1 error, l2 error, PSNR and SSIM comparison of different GAN models on our dataset.

Models	l1 Error	l2 Error	PSNR	SSIM
AC-GAN	13.9%	6.3%	30.73	0.8762
WGAN	12.8%	5.1%	32.61	0.9135
WGAN-GP	12.6%	5.4%	31.42	0.9172
InfoGAN	11.9%	5.7%	31.67	0.9288
WGAN-GP-Info	12.3%	5.2%	31.89	0.9061
DCGAN	12.7%	6.1%	32.77	0.9258
CGAN	14.3%	6.7%	32.33	0.9378
WGAN-GP-AC	9.8%	4.2%	36.71	0.9616

**Table 5:** Precision and recall value for different classification models using original and synthetic datasets.

Classification Models	Original Data Precision	Recall	Synthetic Data Precision	Recall
InceptionV3	0.93	0.92	0.95	0.96
ResNet	0.87	0.89	0.9	0.91
VGG16	0.93	0.9	0.94	0.93
CNN	0.86	0.88	0.89	0.91
Xception	0.88	0.89	0.92	0.92
VGG19	0.91	0.91	0.94	0.96

**Table 6:** Precision and recall value for different classification models using augmentation methods and combination of original and synthetic data.

Classification	Augmention-1		Augmentation-2		Augmentation-3		Original + Synthetic	
Models	Precision	Recall	Precision	Recall	Precision	Recall	Precision	Recall
InceptionV3	0.94	0.93	0.93	0.92	0.94	0.94	0.93	0.94
ResNet	0.88	0.87	0.89	0.88	0.86	0.85	0.89	0.90
VGG16	0.91	0.92	0.90	0.89	0.90	0.90	0.92	0.90
CNN	0.87	0.88	0.86	0.85	0.85	0.84	0.87	0.89
Xception	0.89	0.87	0.87	0.86	0.90	0.89	0.89	0.90
VGG19	0.90	0.88	0.89	0.88	0.90	0.91	0.92	0.93

## Data Availability

Not applicable.
